# Gastroprotective effects of both aqueous and ethanolic extracts of *Lemon verbena* leaves against indomethacin-induced gastric ulcer in rats

**DOI:** 10.22038/ijbms.2020.44341.10377

**Published:** 2020-12

**Authors:** Habibeh Mashayekhi-sardoo, Bibi Marjan Razavi, Maryam Ekhtiari, Negar Kheradmand, Mohsen Imenshahidi

**Affiliations:** 1 Department of Pharmacodynamics and Toxicology, School of Pharmacy, Mashhad University of Medical Sciences, Mashhad, Iran; 2 Targeted Drug Delivery Research Center, School of Pharmacy, Mashhad University of Medical Sciences, Mashhad, Iran; 3 Pharmaceutical Research Center, Institute of Pharmaceutical Technology, Mashhad University of Medical Sciences, Mashhad, Iran

**Keywords:** Indomethacin, Lemon verbena, Lippia citriodora, Gastric ulcer, NSAIDs

## Abstract

**Objective(s):**

Regarding *Lemon verbena* gastroprotective effects, we investigated the protective effects of *Lemon verbena *extracts on reducing gastric ulcer induced by indomethacin.

**Materials and Methods:**

Rats received aqueous and ethanolic extracts of *Lemon verbena *(50, 100, and 200 mg/kg), zileuton (100 mg/kg), montelukast (10 mg/kg), or 1% Tween 80 in presence or absence of indomethacin (100 mg/kg).

**Results:**

Indomethacin produced stomach ulcer and increased neutrophils percentage and MDA level compared with the control group (*P<*0.001). Co-administration of indomethacin and zileuton, montelukast and ethanolic (200 mg/kg) (*P<*0.001), aqueous extract (200 mg/kg) (*P<*0.05) reduced ulcer compared with the indomethacin group (*P<*0.001). Ethanolic extracts (100 and 200 mg/kg) and aqueous extract (200 mg/kg) reduced the MDA level (*P<*0.001). Ethanolic (50, 100, and 200 mg/kg) and aqueous extracts (200 mg/kg) significantly decreased neutrophils percentage compared with the indomethacin group (*P<*0.001).

**Conclusion:**

Aqueous and particularly ethanolic extracts of *Lemon verbena *have protective effects on indomethacin-induced gastric ulcers.

## Introduction

Gastric ulcer is a stomach lining disease featuring common symptoms like vomiting, burning, dull abdominal pain, fever, weight loss, poor oral tolerance, stenosis, perforation, and gastric bleeding (1). Many risk factors, such as *Helicobacter pylori* infection, Nonsteroidal anti-inflammatory drugs (NSAIDs), Acetylsalicylic acid (*Aspirin*, ASA), and alcohol intake play a part in the pathogenesis of gastric ulcers (2-5). 

The main mechanism for NSAIDs damage to gastroduodenal mucosa involves systemic prevention of expressed cyclooxygenase 1 (COX-1)-derived prostaglandins (6, 7). COX inhibition by NSAIDs enhances the synthesis of leukotrienes (LTs) that occur by shunting the arachidonic acid metabolism towards the 5-lipoxygenase (5-LOX) pathway. Cysteinyl leukotrienes (CysLTs), such as LTC4, LTD4, and LTE4 are the main proinflammatory lipid mediators and metabolites of the 5-LOX pathway (8). LTB4 is an important factor in several neutrophile activities like adherence and chemotaxis (9). Additionally, LTs are involved in gastric mucosal complications by triggering tissue ischemia and inflammation (10). The other mechanisms include the generation of reactive oxygen species (ROS) and nitric oxide (NO), the beginning of lipid peroxidation, and penetration of neutrophils secondary to the generation of inflammatory factors. Indomethacin as a beneficial strong NSAID has been considered a drug of choice to induce gastric ulcers (11, 12). Indomethacin changes the arachidonic acid metabolism in neutrophils, through inhibition of COX pathways which directs this metabolism to the 5-LOX pathway, leading to an increase in LT levels (13, 14). This drug through a neutrophil-dependent process, accumulation and neutrophil adhesion, and inducible nitric oxide synthase (iNOS) as a producer of NO, initiates and advances gastric damages (15-17). 

In recent years, the gastroprotective effects of various plants and their secondary metabolites including *Syzygium cumini* (L.) Skeels, *Rosaceae Malus sp*. (Apple), *Persea Americana Mill*. (Avocado), and *Centella Asiatica* (Gotu kola) were reported (18-22). In this context, *Lippia citriodora* known as *Lemon verbena* is a popular medicinal herb in South America and the Mediterranean region (23), and possesses antioxidant (24), gastroprotective (25), and anti-inflammatory (26) properties. *Lemon verbena *extract through its polyphenolic compounds such as Verbascoside inhibits the MPO and free radicals production in neutrophils and is an effective antioxidant and LOX inhibitor (23, 27, 28).

Therefore, this study aimed to evaluate the gastroprotective effect of both aqueous and ethanolic extracts of *Lemon verbena *on the indomethacin-induced gastric ulcers in comparison with two inhibitors of 5-LOX (zileuton and montelukast) as positive controls in Wistar rats.

## Materials and Methods

***Animals***

A total of 72 adult male Wistar rats (220-250 g, 6-7 weeks old) obtained from the Animals Laboratory of Mashhad University of Medical Sciences, Mashhad, Iran, were used in this study. Animals were housed at the Laboratory Animal Facilities of the School of Pharmacy, Mashhad University of medical sciences, Mashhad, Iran (12 hr light/dark cycle, humidity 60±10%, and temperature 25±2 ^°^C). Access to food and water was forbidden for 4 hr before starting the experiment. The animals were allowed adapting to the place condition for 1 week before starting the experiment. All the animal protocols were approved by the Ethical Committee for Animal Experimentation at the School of Pharmacy, Mashhad University of Medical Sciences (code: 922688).

***Chemicals and drugs***

Tween 80 was purchased from Merck Co. (Germany). Indomethacin was obtained from Behdashkar (C.A.S: 53-86-1) (Tehran, Iran). Zileuton was obtained from Cayman (C.A.S: 111406-87-2) (Ann Arbor, Michigan). Montelukast was purchased from Sobhan Darou (C.A.S: 151767-02-1) (Tehran, Iran). Other chemicals were of analytical grade.

***Plant material***

The fresh leaves of *Lemon verbena *were collected from Karaj City, Iran. A voucher specimen was authenticated and deposited in the Herbarium of School of Pharmacy, Mashhad University of Medical Sciences.

***Design of the studied groups***

Rats were divided into 12 independent groups (n=6 per group), treated as detailed in Table 1.

***Extract preparation***

In brief, 100 g of the powdered dried *Lemon verbena *was macerated in 1000 ml of boiling distilled water for the aqueous extract preparation and in 1000 ml of 96% ethanol for the ethanolic extract preparation for 48 hr. After that, the green remaining extracts were smoothed once with gauze and cotton and again with filter paper. The filtered solutions were transferred to Round-bottom balloons. The extracts were separately evaporated by Rotator-Evaporator to remove the solvent completely. After complete removal of the solvents, the extracts were frozen at -20 ^°^C. Then, the balloons were attached to a freeze dryer and a green dried extract powder was yielded. The residues were covered with aluminum foil and kept at a temperature of -20 ^°^C (29). 

***Gastric ulcer induction***

A total of 54 rats received a single dose of 100 mg/kg indomethacin (dissolved in 0.1% tween 80) for half an hour after protective agent administrations in the protective groups and 4 hr after lack of access to food in the groups that only received indomethacin (14).

***Determination of macroscopic gastric ulcer score***

On the experiment day, the rats did not receive water and food for 4 hr. Extracts and control were administered intraperitoneally to rats half an hour before the oral administration of indomethacin. Four hours after the administrations of indomethacin, rats were killed and gastric tissue was removed and cut off from the posterior wall and washed in normal saline to remove the contents. Gastric ulcer index according to the severity of stomach damage was scored as below: 

1= Petechia; 5= lesion size 1-3 mm; 10= lesion size up to 3 mm

Finally, each stomach was placed in a separate tube and kept at the temperature of -80 ^°^C for other experiments (14, 30).

***Measurement of lipid peroxidation indices (Malondialdehyde (MDA) level)***

First, 10% homogenate of the stomach tissue was mixed with 1.15% potassium chloride in a container of ice, and then 0.5 ml of it mixed with 3 ml of phosphoric acid 1% and 1 ml of Thiobarbituric acid (TBA) 0.6 % in some tubes. The tubes were placed in boiling water for 45 min, and after cooling, 4 ml of n-butanol was added to each tube to remove the colored complex, and after that, they were vortexed for 1 min. Finally, the tubes were placed inside the centrifuge at 4000 rpm for 20 min at 4 ^°^C. After completion of centrifugation, the supernatant was separated and the absorbance was read with a spectrophotometer (Jenway 6105 UV/vis, England) at 532 nm. The standard curve was drawn up in a concentration range of 0-100 nmol/ml for MDA and the concentrations were calculated and reported as nmol/g tissue value (31).

***Calculating the percentage of neutrophils in the blood***

Sixty minutes after administration of indomethacin, blood samples were collected from rat eyes and transferred to heparin vials and sent to the laboratory to determine neutrophil counts (14, 30).

***Statistical Analysis***

Statistical analysis was carried out using the Prism 6 software package and the tests including one-way ANOVA test and *post hoc* Tukey-Kramer test were used for comparison of MDA levels and percentage of neutrophils in the blood. Moreover, the scores of gastric ulcers with Kruskal–Wallis test were evaluated and the results were shown via the median. Data were shown as mean values±standard deviation (SD). The statistical differences are significant if the *P-value* ≤ 0.05.

## Results

***Gastric ulcer index***

**Figure 1:**
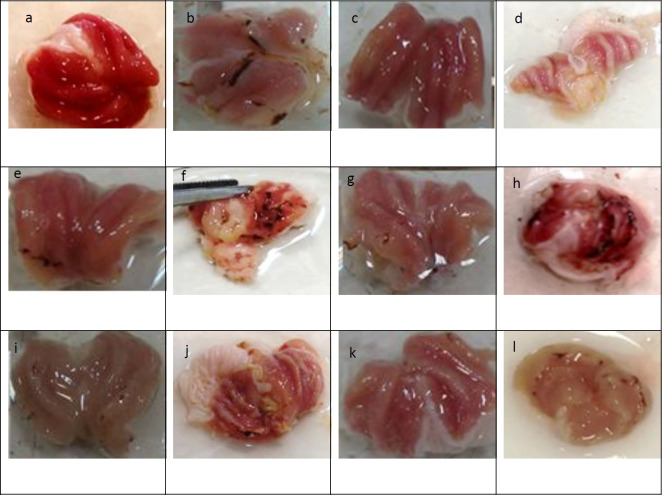
Macroscopic observation of the gastric ulcers, (a). 0.1% Tween 80, (b). indomethacin (100 mg/kg), (c). ethanolic extract of *Lemon verbena* (200 mg/kg), (d). aqueous extract of *Lemon verbena *(200 mg/kg), (e). indomethacin (100 mg/kg)+ethanolic extract of *Lemon verbena *(50 mg/kg), (f). indomethacin (100 mg/kg)+aqueous extract of *Lemon verbena *(50 mg/kg), (g). indomethacin (100 mg/kg)+ethanolic extract of *Lemon verbena *(100 mg/kg), (h). indomethacin (100 mg/kg)+aqueous extract of *Lemon verbena *(100 mg/kg), (i). indomethacin (100 mg/kg)+ethanolic extract of *Lemon verbena *(200 mg/kg), (j). indomethacin (100 mg/kg)+aqueous extract of *Lemon verbena *(200 mg/kg), (k). indomethacin (100 mg/kg)+zileuton (100 mg/kg), (l). indomethacin (100 mg/kg)+montelukast (10 mg/kg)

**Figure 2:**
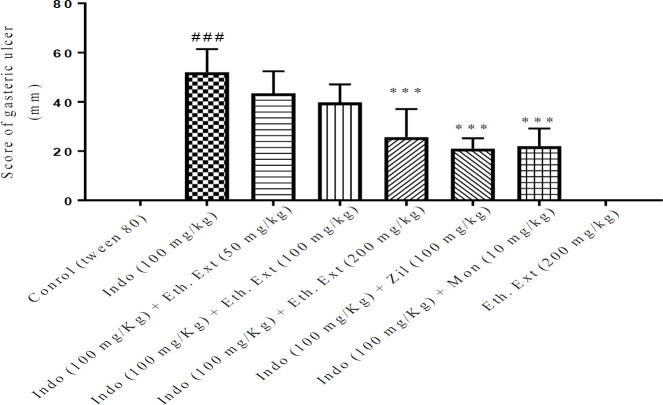
Effects of ethanolic extract of *Lemon verbena*, zileuton, and montelukast on the gastric ulcers induced by indomethacin. Ethanolic extracts of *Lemon verbena *were administered intraperitoneally (IP) and zileuton and montelukast by gavage. The number of rats in each group was 6. The data were reported as median±IQR after analysis by the Kruskal–Wallis test. Statistical analyses showed: *** *P<*0.001 for comparison with the indomethacin group and ^###^ *P<*0.001 for comparison with the control group

**Figure 3:**
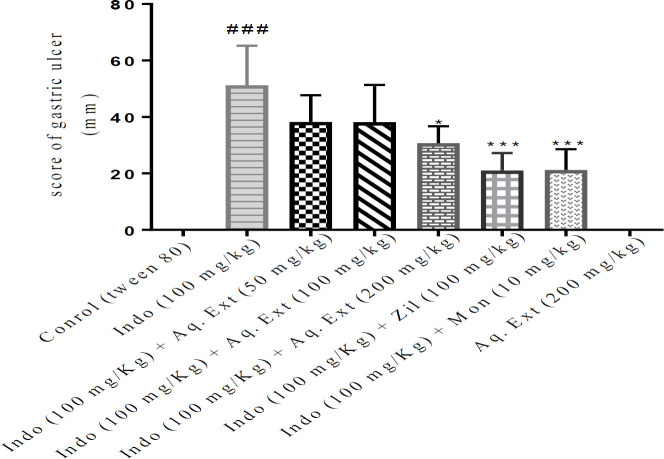
Effects of aqueous extract of *Lemon verbena*, zileuton, and montelukast on the gastric ulcers induced by indomethacin. Aqueous extracts of *Lemon verbena *were administered intraperitoneally (IP) and zileuton and montelukast by gavage. The number of rats in each group was 6. The data were reported as median±IQR after analysis by Kruskal–Wallis test. Statistical analyses showed: *** *P<*0.001 and * *P<*0.05 for comparison with indomethacin group and ^###^ *P<*0.001 for comparison with the control group

The gastric ulcer index was determined by evaluating the ulcer score on the stomach mucosal wall. Indomethacin administration considerably increased the ulcer index and pathological score values in the stomach tissue compared with the control group (*P*<0.001).The ethanolic extract of *Lemon verbena *(200 mg/kg), zileuton, and montelukast significantly reduced the ulcer index (*P*<0.001). The aqueous extract of *Lemon verbena *(200 mg/kg) also resulted in a substantial reduction in the ulcer index compared with the indomethacin group (*P*<0.05). A comparison of the protective effect of high doses of aqueous and ethanolic extracts of *Lemon verbena *with zileuton and montelukast did not show any significant difference. The macroscopic observations and scores of the gastric ulcers were shown in Figures 1-3.

***Effect of indomethacin and extracts of Lemon verbena on lipid peroxidation induced by indomethacin in gastric tissue***

**Figure 4:**
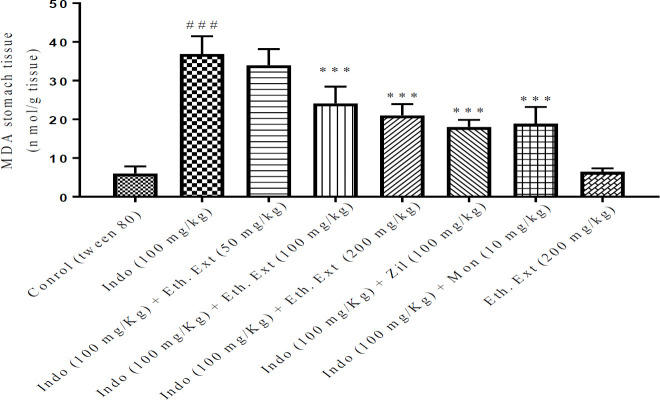
Effects of ethanolic extract of *Lemon verbena*, zileuton, montelukast on MDA levels in the stomach tissue after gastric ulcer induced by indomethacin. ethanolic extracts of *Lemon verbena *were administered intraperitoneally (IP) and zileuton and montelukast by gavage. The number of rats in each group was 6. the data were analyzed by the *post hoc* Tukey-Kramer test. Statistical analyses showed: *** *P<*0.001 for comparison with the indomethacin group and ^###^ *P<*0.001 for comparison with the control group

**Figure 5:**
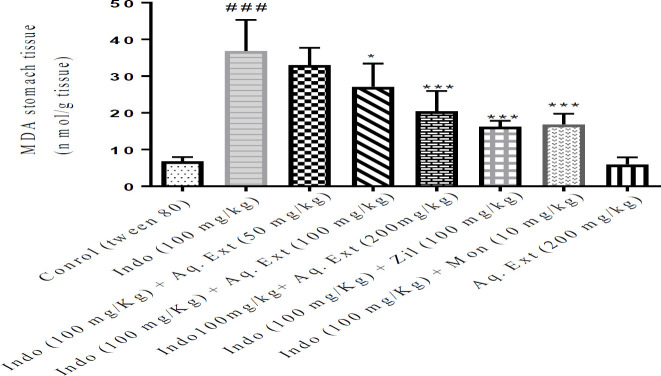
Effects of aqueous extract of *Lemon verbena*, zileuton, montelukast on MDA levels in the stomach tissue after gastric ulcers induced by indomethacin. Aqueous extracts of *Lemon verbena *were administered intraperitoneally (IP) and zileuton and montelukast by gavage. The number of rats in each group was 6. The data were analyzed by the *post hoc* Tukey-Kramer test. Statistical analyses showed: *** *P<*0.001 and * *P<*0.05 for comparison with indomethacin group and ^### ^*P<*0.001 for comparison with the control group

In the gastric tissue, the MDA level in the indomethacin treatment group was remarkably higher when compared with the control group (*P*<0.001). Meanwhile, pretreatment with ethanolic extract of *Lemon verbena *(100 and 200 mg/kg), the aqueous extract of *Lemon verbena *(200 mg/kg), zileuton (100 mg/kg), and montelukast (10 mg/kg) has shown a marked decrease in MDA amounts of the gastric tissue in comparison with the indomethacin alone group (*P*<0.001). No significant difference was found between ethanolic and aqueous extracts of *Lemon verbena *(200 mg/kg) compared with zileuton (100 mg/kg) and montelukast (10 mg/kg) groups. Figures 4 and 5 present the summary of the effects of both ethanolic and aqueous extracts of *Lemon* verbena, zileuton, and montelukast on MDA levels in the stomach tissue after indomethacin-induced gastric ulcer.

***Percentage of neutrophils in the Blood***

**Figure 6:**
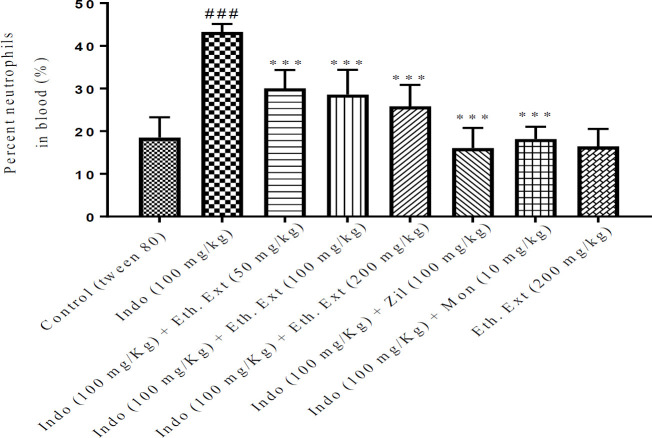
Effects of ethanolic extract of *Lemon verbena*, zileuton, montelukast on the percentage of blood neutrophils after gastric ulcers induced by indomethacin. Ethanolic extracts of *Lemon verbena *were administered intraperitoneally (IP) and zileuton and montelukast by gavage. The number of rats in each group was 6. The data were analyzed by the *post hoc* Tukey-Kramer test. Statistical analyses were shown: *** *P<*0.001 for comparison with indomethacin group and ^### ^*P<*0.001 for comparison with the control group

**Figure 7:**
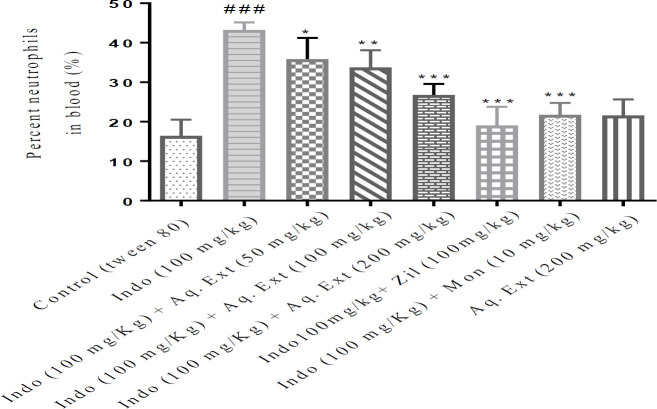
Effects of aqueous extract of *Lemon verbena*, zileuton, montelukast on the percentage of blood neutrophils after gastric ulcers induced by indomethacin. Aqueous extracts of *Lemon verbena *were administered intraperitoneally (IP) and zileuton and montelukast by gavage. The number of rats in each group was 6. The data were analyzed by the *post hoc* Tukey-Kramer test. Statistical analyses showed: *** *P<*0.001, ** *P<*0.01 and * *P<*0.05 for comparison with the indomethacin group and ^###^ *P<*0.001 for comparison with the control group

Exposure to indomethacin remarkably increased the percentage of blood neutrophils compared with the control group (*P*<0.001). Pretreatment with ethanolic extract of *Lemon verbena *at three doses of 50, 100, and 200 mg/kg, the aqueous extract of *Lemon verbena *(200 mg/kg), zileuton (100 mg/kg), and montelukast (10 mg/kg) significantly decreased the blood neutrophils in comparison with the indomethacin group (*P*<0.001). Aqueous extract of *Lemon verbena *at doses of 100 mg/kg and 50 mg/kg contributed to a notable decrease in blood neutrophils compared with the indomethacin group (*P*<0.01 and *P*<0.05, respectively). The results of aqueous and ethanolic extracts of *Lemon verbena*, zileuton, and montelukast on the percentage of blood neutrophils after gastric ulcers induced by indomethacin are shown in Figures 6 and 7.

**Table 1:** Design of the experimental groups

**groups**	**pretreatment**	**main treatment**
group 1		a single dose of 0.1% tween 80 as a negative control
		1 ml, oral
group 2		a single dose of indomethacin
		100 mg/kg, oral
group 3	a single dose of ethanolic extract of *lemon verbena*	
	200 mg/kg, IP^a^	
group 4	a single dose of ethanolic extract of *lemon verbena*	a single dose of indomethacin
	50 mg/kg, IP	100 mg/kg, oral
group 5	a single dose of ethanolic extract of *lemon verbena*	a single dose of indomethacin
	100 mg/kg, IP	100 mg/kg, oral
group 6	a single dose of ethanolic extract of *lemon verbena*	a single dose of indomethacin
	200 mg/kg, IP	100 mg/kg, oral
group 7	a single dose of aqueous extract of *lemon verbena*	
	200 mg/kg, IP	
group 8	a single dose of aqueous extract of *lemon verbena*	a single dose of indomethacin
	50 mg/kg, IP	100 mg/kg, oral
group 9	a single dose of aqueous extract of *lemon verbena*	a single dose of indomethacin
	100 mg/kg, IP	100 mg/kg, oral
group 10	a single dose of aqueous extract of *lemon verbena*	a single dose of indomethacin
	200 mg/kg, IP	100 mg/kg, oral
group 11	a single dose of zileuton as a positive control	a single dose of indomethacin
	100 mg/kg, oral	100 mg/kg, oral
group 12	a single dose of montelukast as a positive control	a single dose of indomethacin
	10 mg/kg, oral	100 mg/kg, oral

^a^ intraperitoneal injection

eth. ext: ethanolic extract, zil: zileuton, mon: montelukast, indo: indomethacin

aq. ext: aqueous extract, zil: zileuton, mon: montelukast, indo: indomethacin

eth. ext: ethanolic extract, zil: zileuton, mon: montelukast, indo: indomethacin

aq. ext: aqueous extract, zil: zileuton, mon: montelukast, indo: indomethacin

eth. ext: ethanolic extract, zil: zileuton, mon: montelukast, indo: indomethacin

aq. ext: aqueous extract, zil: zileuton, mon: montelukast, indo: indomethacin

## Discussion

The present study demonstrated the gastroprotective effect of three different doses of aqueous and ethanolic extracts of *Lemon verbena *on indomethacin-induced gastric ulcers in rats. Indomethacin produced obvious macroscopic stomach ulcers compared with the control group. It also increased the percentage of neutrophils in the blood and increased MDA levels compared with the control group. The ethanolic extract of *Lemon verbena *(50, 100, and 200 mg/kg) and the aqueous extract of *Lemon verbena *(200 mg/kg), similar to zileuton and montelukast, significantly decreased the percentage of neutrophils in the blood that indomethacin had elevated. Despite co-administration of indomethacin and zileuton, montelukast and ethanolic extract of *Lemon verbena *(200 mg/kg) remarkably reduced stomach ulcers compared with the indomethacin (100 mg/kg) alone group (*P*<0.001), 200 mg/kg of aqueous extract (*P*<0.05) less effectively than 200 mg/kg of the ethanolic extract could reduce stomach ulcers compared with the indomethacin alone group. The ethanolic extract of *Lemon verbena *at doses of 100 and 200 mg/kg and the aqueous extract of *Lemon verbena *at the dose of 200 mg/kg could decrease the MDA levels in gastric tissue. Furthermore, zileuton and montelukast also diminished the MDA contents in the stomach tissue.

Administration of NSAIDs, especially indomethacin leads to microscopic and macroscopic wounds in animals that resemble the gastric ulcers in humans (32). In our study similar to previous studies oral administration of indomethacin at a dose of 100 mg/kg induced an obvious macroscopic gastric ulcers in rats (14, 33). Inhibiting the COX enzyme by NSAIDs leading to a decrease in mucosal synthesis and bicarbonate secretion, and an increase in leukocyte accumulation is the main cause of gastric ulcers (14). Several studies revealed indomethacin, through the prevention of PGs synthesis in the arachidonic acid metabolism pathway, stimulates the 5-LOX enzyme leading to elevated LT’s amounts (13, 34). On the other hand, LTB (4) 12-HD/PGR is a crucial enzyme for eicosanoid inactivation and acts an important part in the metabolism of PGs E and F, and LTB4. This enzyme is a potent chemoattractant agent for inflammatory leukocytes and 15-oxo-lipoxin A (4) (15-oxo-LXA (4)). Some NSAIDs like indomethacin apart from inhibition of COX can act as LTB (4) 12-HD/PGR inhibitor (35, 36) and thus cause an increase in LT’s levels. Some studies have found that LTB4, LTC4, or LTD4 are the main factors in the development of vascular damage and mucosal lesions in the gastric tissue (37-39). One attempt has been made to investigate the protective impact of 5-LOX inhibitors and LT antagonists on the development of gastric ulcers after indomethacin intake. The results of the study highlighted that overproduction of metabolites of the 5-LOX pathway plays an important role in the development of gastric injury, and 5-LOX inhibitors and LTs antagonists’ agents reverse these gastric damages induced by indomethacin (40). The most important sources of LTs are neutrophils, which are more effective in the pathogenesis of gastrointestinal tract ulcers induced by NSAIDs, especially indomethacin (30). For this reason, we carried out an experiment to investigate the effect of indomethacin on neutrophil counts. The main functions of LTs are the invitation of neutrophils and chemokines and causing neutrophil adhesion to epithelial cells. Moreover, LTs activate neutrophils to release mediators, ultimately lending to degranulation (41). Activated neutrophils exhibited microvascular disturbance and then resulted in gastric erosions following NSAIDs intake (42). Elevated LTB4 concentrations trigger chemotaxis, adhesion, and degranulation of neutrophils. These processes stimulate gastric mucosal injuries (43). The previous studies have proven that gastrointestinal ulceration resulting from indomethacin intake are due to increased LTs production followed by an increment in neutrophil infiltration and gastric MPO activity (14, 41, 44). Singh *et.al* in 2005 (30), expressed that pretreatment of leukoflone as an inhibitor of COX 1/2-5-lipoxygenase created gastroprotective functions against high amounts of produced LB4 following great infiltration of neutrophils in both rat and mice blood caused by indomethacin. Our study showed that both extracts of *Lemon verbena,* similar to montelukast and zileuton, dramatically reduced the gastric lesion score; inhibition of the 5-LOX pathway by these protective agents proved the feasible role of LTs in gastric damage caused by indomethacin. In a study, montelukast and curcumin (inhibitor of COX and LOX) protected the gastric tract against the damages caused by indomethacin. Besides, montelukast and curcumin reduced neutrophil adherence by a reduction in the production of LTs compared with the indomethacin group (14). Dengiz GO *et al.* (45) revealed montelukast through amelioration of oxidative damage and MPO function can play a gastroprotective role against indomethacin-induced gastric ulcers. The other investigation showed that montelukast is capable of decreasing the MPO function in neutrophils, prevention of LTs synthesis, and stomach mucosal permeability to involved ions and local increment in the PGs production after gastric ulcers induced by indomethacin. Furthermore, the lipid peroxidation process influences gastric ulcers through MDA production (46), hence the high levels of MDA indicate an increase in the levels of free radicals (47). It is supported by the results of the study, that the administration of indomethacin to rats resulted in a marked increase in MDA levels compared with controls. 

*Lemon verbena *owing to having polyphenol compounds such as verbascoside and flavones exhibit strong antioxidant activity and lipoxygenase inhibition (24, 48). Verbascoside is the main water-soluble phenylpropanoid glycoside component of *L. citriodora* extracts (24, 49). The verbascoside in equimolar mixtures is a potent inhibitor of oxidative stress and because of catechol groups is capable of scavenger activity and also with sugar residue shows solubility and reducing effects (50, 51). A study (48), after evaluation of various extraction procedures reported that ethanolic extract of *Lemon verbena *exhibited a higher capacity of recovery of organic compounds in comparison with the aqueous extract of *Lemon verbena*. Notably, the ethanolic extract of *Lemon verbena *can purify verbascoside and its analogs. 

It appears that the present study confirms previous findings and contributes additional evidence that suggests ethanolic extracts of *Lemon verbena *due to higher recovery of verbascoside, as the main ant potent compound of *lemon verbena,* highlighted more gastroprotection than the aqueous extract of *Lemon verbena *against indomethacin-induced gastric ulcer.

Daily administration of *Lemon verbena *extract (water: methanol 1:1, containing 10% verbascoside) to athletes leads to a significant decrease in the levels of MDA in neutrophils and protected these cells against oxidative stress lesion compared with the control group (52). Furthermore, the aqueous extract of *L. citriodora* with an unknown mechanism more strongly healed gastric ulcers induced by ethanol in rats in comparison with ranitidine (53). *L. citriodora* owing to verbascoside compound included pharmacological activity showing anti-inflammatory activity by prevention of MPO synthesis in neutrophils and antioxidant defense against free radical productions in all different blood cell types (23). A study (27), revealed that *Lemon verbena *extract, because of containing verbascoside as a strong radical scavenger, exhibited potent antioxidant activity such as decrement in MDA levels. A study introduced verbascoside as a noteworthy inhibitor of both 5-LOX and protein kinase C (PKC) apart from capability in free radical scavenging and antioxidant characteristics (54). Verbascoside possessed anti-inflammatory properties as a result of a reduction in superoxide radical generation and GPx function, and consequently decrease in iNOS activity (55).

To sum up, this experiment substantiates strong gastroprotection of *Lemon verbena *against indomethacin-induced gastric ulcers with comparable efficacy to CysLT inhibitors such as zileuton and montelukast. The prospective usage of *Lemon verbena *as a protective compound versus gastric ulcer remains an open area for future research.

## Conclusion

In brief, this is the first study to evaluate the protective effects of *Lemon verbena *against the indomethacin-induced gastric ulcers in rats. The results of this study showed that intraperitoneal administration of aqueous and ethanolic extracts of *Lemon verbena *at a dose of 200 mg/kg has a protective effect on the development of gastric ulcers induced by indomethacin possibly by inhibiting the LOX enzyme or inhibiting LTs receptors and lipid peroxidation. Concerning the evidence on protective effects of the extracts of *Lemon verbena *in reducing the gastrointestinal damage induced by indomethacin, future experiments are needed to investigate the protective effects of the active ingredients of *Lemon verbena *on healing the gastric ulcers and to explore the involved mechanisms regarding protective effects of *Lemon verbena *against gastrointestinal damage caused by indomethacin.
